# The Stool Volatile Metabolome of Pre-Term Babies

**DOI:** 10.3390/molecules26113341

**Published:** 2021-06-02

**Authors:** Alessandra Frau, Lauren Lett, Rachael Slater, Gregory R. Young, Christopher J. Stewart, Janet Berrington, David M. Hughes, Nicholas Embleton, Chris Probert

**Affiliations:** 1 Institute of Systems, Molecular and Integrative Biology, University of Liverpool, Crown Street, Liverpool L69 3GE, UK; llett@liverpool.ac.uk (L.L.); rsh14@liverpool.ac.uk (R.S.); mdcsjp@liverpool.ac.uk (C.P.); 2 Hub for Biotechnology in the Built Environment, Northumbria University, Newcastle upon Tyne NE1 8ST, UK; gregory.young@northumbria.ac.uk; 3 Translational and Clinical Research Institute, Faculty of Medical Sciences, Newcastle University, Newcastle upon Tyne NE1 8ST, UK; Christopher.Stewart@newcastle.ac.uk (C.J.S.); j.e.berrington@newcastle.ac.uk (J.B.); 4 Department of Neonatology, Newcastle upon Tyne Hospitals NHS Foundation Trust, Newcastle upon Tyne NE1 8ST, UK; nicholas.embleton@newcastle.ac.uk; 5 Department of Health Data Science, University of Liverpool, Liverpool, Merseyside L69 3GA, UK; dmhughes@liverpool.ac.uk; 6 Population Health Sciences Institute, Newcastle University, Newcastle upon Tyne NE1 8ST, UK

**Keywords:** metabolome, feces, neonates, fermentation, protein, carbohydrate, short chain fatty acid

## Abstract

The fecal metabolome in early life has seldom been studied. We investigated its evolution in pre-term babies during their first weeks of life. Multiple (n = 152) stool samples were studied from 51 babies, all <32 weeks gestation. Volatile organic compounds (VOCs) were analyzed by headspace solid phase microextraction gas chromatography mass spectrometry. Data were interpreted using Automated Mass Spectral Deconvolution System (AMDIS) with the National Institute of Standards and Technology (NIST) reference library. Statistical analysis was based on linear mixed modelling, the number of VOCs increased over time; a rise was mainly observed between day 5 and day 10. The shift at day 5 was associated with products of branched-chain fatty acids. Prior to this, the metabolome was dominated by aldehydes and acetic acid. Caesarean delivery showed a modest association with molecules of fungal origin. This study shows how the metabolome changes in early life in pre-term babies. The shift in the metabolome 5 days after delivery coincides with the establishment of enteral feeding and the transition from meconium to feces. Great diversity of metabolites was associated with being fed greater volumes of milk.

## 1. Introduction

The intestinal metabolome is shaped by the interactions between the microbiota and diet. Before birth, mammals ingest amniotic fluid which contains amino acids (notably taurine), some proteins (including growth factors and hormones), phospholipids [1], and, potentially, bacteria [2] and volatile organic compounds, from the mother [3]. Soon after birth, bacteria and other microbes that will eventually form the microbiota begin to colonize the intestine. During the neonatal period, there is a huge switch in the enteral intake from amniotic fluid, to colostrum and then milk, in the majority of babies. Colostrum and milk also contain microbes which may seed to the baby [4,5]. Babies that are born significantly pre-term are cared for in Neonatal Intensive Care Units (NICUs) where they receive expressed colostrum and breast milk, if possible.

It has been proposed that the study of feces from neonates may be useful in the early identification of necrotizing enterocolitis (NEC) [6,7,8] and late onset sepsis (LOS), to which preterm babies are at risk. There is a paucity of research on the metabolome in early life and we hypothesize that disease signals may be obscured as the metabolome is rapidly changing.

Here, we have analyzed the metabolome of a new cohort of preterm babies, who did not develop NEC or late onset sepsis, and explore factors that might have an impact on the metabolome. The paper describes the ‘normal metabolome of the preterm neonate’ as a reference document for others interested in the health of the newborn.

## 2. Results

### 2.1. Patients Demographics

Fifty-one healthy infants (not affected by NEC or LOS), all <32 weeks gestation at birth and participating in both the Enteral LactoFerrin In Neonates (ELFIN) and mechanisms affecting the gut of preterm infants in enteral feeding trials (MAGPIE) [9] studies, were used in this sub-study. A total of 152 samples were analysed (distribution of age and samples shown in Table 1). Of the 51 infants, 46 were twins and 7 were singletons; their key neonatal features are summarised below.

### 2.2. Metabolomic Profile of Stool Samples from Pre-Term Babies

There were 36 volatile organic compounds (VOCs) present in at least 25% of samples (Table 2, Appendix A Table A1). The three short chain fatty acids, acetic acid, propionic acid and butanoic acid, were present in 91%, 53% and 42% of samples, respectively. Aldehydes and alcohols were the largest groups with 6 compounds in each group.

**Figure 1:**
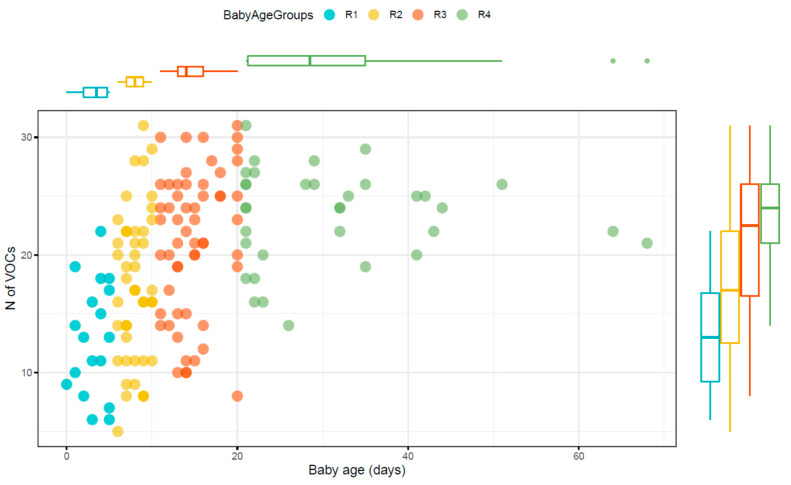
Scatterplot and boxplots to show the number of volatile organic compounds (VOCs) in each of the age groups. Each dot represents a sample (all samples were included, n = 152). (R1 = up to 5 (n = 18), R2 = 6–10 (n = 44), R3 = 11–20 (n = 56), R4 = 21–70 days (n = 34).

We then investigated the impact of the infants’ postnatal age (all samples were included, n = 152). A mixed effect regression model of VOC number per patient and postnatal age (days) showed a significant (*p*-value < 0.0001) increase in VOCs during time of 1.0126 compound per day (95% Wald confidence interval 1.009, 1.016). Subsequentially, samples were grouped by age (Table 3). The number of VOCs was limited in the first 5 days of life (Table 3) (Figure 1). Anova analysis (f-ratio = 16.55624, *p* < 0.00001, post hoc HSD) showed that the number of VOCs was significantly different among the groups: R1 < R2**, R3****, R4**** and R2 < R3*, R4***. R3 and R4 not significantly different. (* 0.05, ** < 0.01, *** < 0.001, **** < 0.0001).

Scrutiny of the data showed that some VOCs were present in the majority of babies in each age group. Others started to appear in the second or third groups. Nine VOCs were present in >66% of samples in first group (1–5 days): 4 were aldehydes—hexanal (100%), heptanal (67%), octanal (78%) and nonanal (67%); 2 others were methylated aldehydes, 2-methylbutyraldehyde (67%) and isovaleraldehyde (89%); the remainder were acetic acid (89%), 2-pentylfuran (72%), and 1-octen-3-ol (67%). These 9 VOCs remained common in the later samples. The second group (6-10 days) had 4 further VOCs that were found on >60% of samples: these were 2-methylbutanoic acid (61%), isovaleric acid (70%), 2,3-butanedione (64%) and 6-methyl-5-hepten-2-one (59%). While acetic acid was common in all 4 groups, propionic acid (range 38–64%) and butanoic acid (11–62%) were not.

**Figure 2:**
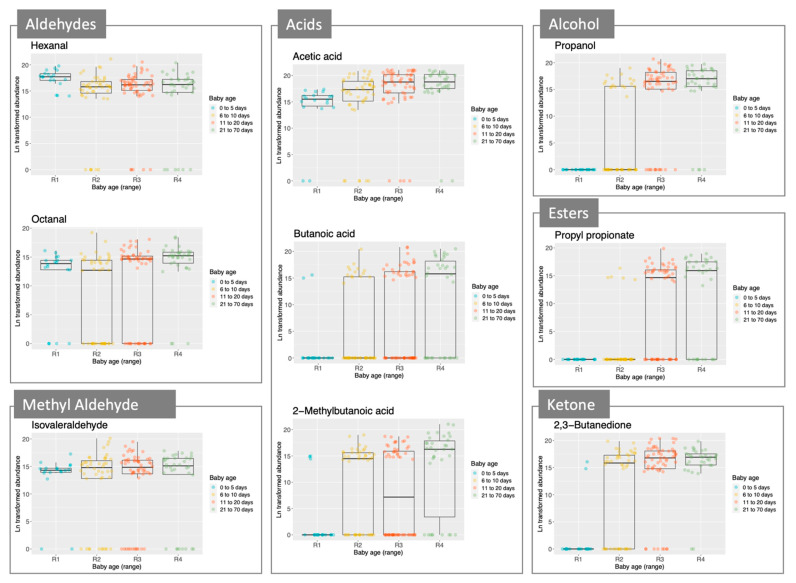
Boxplots for a selection of compounds (abundance/age group). Each boxplot represents a compound, and these are grouped according to the type of molecule (i.e., aldehydes, methyl aldehydes, acids, alcohol, esters, and ketone). All samples were included, n = 152.

In Figure 2, we focus on a selection of compounds, showing that some of these, specifically, aldehydes and acetic acid, were present since birth and others (acids, esters, ketones and alcohol), increased after day 5.

**Figure 3:**
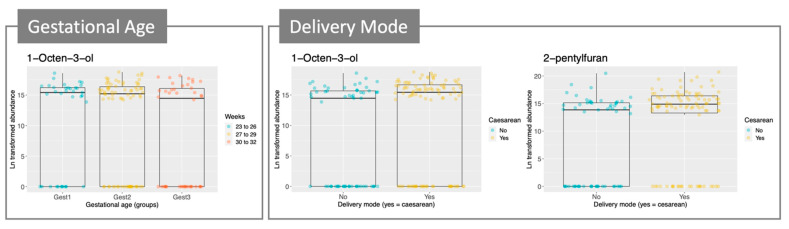
Boxplots for a selection of compounds (abundance/gestational age and delivery mode). Each boxplot represents a compound, and these are grouped according to the variable of interest (gestational age and delivery mode). All samples were included, n = 152.

Linear mixed-effects (LME) analysis was used to identify compounds that changed over time, results are in Table 4. All of these increased over times (positive slope value). Three other factors were considered in the analysis: batch, gestational age (weeks) and delivery mode. Patient ID was a random effect in the LME analysis. Esters were slightly increased over time in babies with a higher gestational age, meanwhile an alcohol and a ketone show a weak increase in babies born earlier during the pregnancy. Interestingly, the alcohol, 1-octen-3-ol (Table 4 and Figure 3), can be related to fungal metabolism [10,11]. This metabolite and 2-pentylfuran, another compound related to fungal metabolism [12], were also slightly increased in babies born by caesarean section (Table 4 and Figure 3). Most of the compounds that showed significant association with delivery mode were increased in babies born by caesarean section, except for ethyl acetate that was increased in babies born by vaginal delivery.

## 3. Discussion

This is the largest study of the fecal metabolome in the neonatal period. Samples from the first few days after birth are characterized by the limited range of VOCs and the predominance of acetic acid and aldehydes. We found that acetic acid was found in the majority of these samples, but propionic acid and butanoic acid were not. Studies on the fermentation of taurine have shown that acetic acid is the most common short-chain fatty acid (SCFA) derived from this amino acid [13]: it is plausible that the taurine-rich amniotic fluid is responsible for this pattern of SCFA in the meconium.

The presence of aldehydes was striking. There were four medium-chain aldehydes (C6–C9) and two further branched aldehydes. Aldehydes are a consequence of lipid peroxidation [14,15]. Branched-chain aldehydes arise from amino acids (for example, leucine and isoleucine [16,17]) and are metabolites of lactic acid bacteria, which are abundant in the vagina and are likely to seed to the neonate during delivery.

There was a steady increase in the range (median 13 to 24, ANOVA *p* < 0.00001) of VOCs in faecal samples during the first few weeks of life. The lack of esters was striking. Esters are common in adult faeces and may arise from foods (as flavours in fruit [18]) but may occur by the condensation of fatty acids and alcohols [19].

The previous study of VOCs in preterm new-borns [6] reported 36 samples were obtained from seven babies over 14 days. The same analytical laboratory methods were used although the present study had more consistent stool weights (80.6 mg (range 32.5–100 mg, SD 12.3 mg) than the earlier one (890 mg, range 300–2400 mg, SD 460 mg). The main difference between these two studies was the temporal sampling employed here: the earlier report did not consider the influence of the age of the babies. As a result, no conclusions could be drawn about the evolution of the metabolome. Costello noted that 7 of the 15 most abundant compounds were aldehydes. Acetone and ethanol were also prevalent. 2-ethylhexanol was also common (97%), but it was considered to be a contaminant arising from plasticware: it was found in 61% of samples in the present study, even though samples were collected into glass vials. The three short chain fatty acids are common in the stool of adults (>95%) [19], each had a low prevalence (<10%) in the Costello study.

The paper reports the evolution of the faecal metabolome in the first weeks of life in preterm babies. There is a marked change that occurs in association with the introduction of first milk feeds. The lack of SCFA in the first week of life suggests they are not a requirement for the intestine in utero or early after birth; their appearance when milk is introduced suggests that the faecal microbiota contains bacteria able to ferment carbohydrates and amino acids to synthesize SCFA.

Gestational age and delivery mode were included in our LME model as these factors are known to influence the gut microbiota of infants. A weak increase in fungal metabolites was observed in babies born earlier during the pregnancy and delivered by caesarean section. In full-term infants, mode of delivery is known to influence the microbiota and it has been shown that babies born by caesarean delivery are more susceptible to being colonized by opportunistic pathogen acquired from the hospital environment rather than commensal bacteria that are transmitted by the mother during vaginal delivery [20]. This effect may increase in babies spending a long time in NICU and may explain the increase in signal of fungal volatile (1-octen-3-ol and 2-pentylfuran), as yeasts may colonize the gut in an opportunistic fashion and NICU are a source of yeasts [21]. Similarly, earlier preterm babies showed a weak increase in fungal metabolites. A recent study on interkingdom relationships (bacteria, fungi and archaea) on preterm infants [22] found a defined succession of bacteria genera, however the evolution of the fungal community was less predictable. They found a negative correlation between fungal and bacterial load, and that *Candida* colonization was inhibited by *Staphylococcus*, a pioneer in the establishment of gut microbiota in early life [23].

## 4. Materials and Methods

### 4.1. Patients

Patients in this sub-study were part of a large cohort recruited to the MAGPIE study. This study focuses on the children without necrotising enterocolitis or late onset sepsis, who gave a least two stool samples during the first 70 days of life. The overarching study was the ELFIN study. Preterm infants at one of 12 participating NHS hospital trusts (13 separate NICUs) were eligible if they met enrolment criteria for ELFIN which included preterm infants < 32 weeks gestation and <72 h postnatal age. Potential infants meeting the eligibility criteria for MAGPIE were identified and recruited by the local healthcare team. Parents were approached for written informed consent after they had received a verbal and written explanation of MAGPIE. The study protocol was approved by East Midlands—Nottingham 2 Research Ethics Committee 16/EM/0042.

### 4.2. Extraction of VOCs

Faecal samples collected in glass vials and stored at −80 °C in Newcastle for up to 12 months, before shipping to the Liverpool laboratory, on dry ice, and being stored at −20 °C again. Prior to analysis, samples were weighed, and aliquots transferred to 10 mL glass headspace vials with magnetic septum caps (Sigma-Aldrich, Dorset, UK) in a hood: a mean of 80.6 mg stool (SD 12.3 mg) was used for the analysis. During aliquoting an empty vial remained unsealed in the hood to collect circulating air, later this was then re-sealed in the hood and was stored with the prepared samples. These air samples were analysed alongside the samples to determine whether there were contaminants in the air when the samples were aliquoted.

Volatile organic compound analysis was performed using gas-chromatography mass-spectrometry on a PerkinElmer Clarus 500 GC-MS quadrupole benchtop system (Beaconsfield, UK) and Combi PAL auto-sampler (CTC Analytics, Zwingen, Switzerland). VOCs were extracted using solid phase micro-extraction with a divinylbenzene-carboxen-polydimethylsiloxane (DVB-CAR-PDMS) (Sigma-Aldrich, Dorset, UK) coated fibre, otherwise the protocol and GC-MS conditions were the same as published by Reade et al. (2014) [24]. Samples were heated to 60 °C for 30 min at prior to fibre exposure, the fibre was exposed to the headspace gases at 60 °C for 20 min, then thermally desorbed for 5 min at 220 °C.

The GC column used was a 60 m Zebron ZB-624 (inner diameter 0.25 mm, length 60 m, film thickness 1.4 μm (Phenomenex, Macclesfield, UK). The carrier gas used was 99.996% pure helium (BOC, Sheffield, UK) which was passed through a helium purification system, Excelasorb^™^ (Supelco, Bellefonte, PA, USA) at 1 mL/min. The initial temperature of the GC oven was set at 40°C and held for 2 min before increasing to 220 °C at a rate of 5 °C/min and held for 4 min with a total run time of 41 min. The MS was operated in electron impact ionization EI + mode, scanning from 10 to 300 *m*/*z* with an interscan delay of 0.1 s and a resolution of 1000 at FWHM (Full Width at Half Maximum). Samples were run in two batches, the first batch had 36 samples and the second 116.

### 4.3. Downstream Data Processing and Analysis

The GC-MS data were processed as CDF files using the Automated Mass Spectral Deconvolution and Identification System software (AMDIS, version 2.73, 2017, Gaithersburg, MD, USA), the NIST mass spectral library ((version 2.0, 2011 purchased from PerkinElmer, Beaconsfield, UK) and the R package Metab [25]. AMDIS and NIST software were used to build a compound library; VOCs were added based on a match criterion of greater than 700, then a probability of a true match (greater than 70%) and finally inspection of fragment patterns. This compound library is then used, with AMDIS, and was applied to deconvolute chromatograms and identifying metabolites. VOCs were named as common names, moreover, the International Union of Pure and Applied Chemistry (IUPAC) [26] names along with PubChem CID number are provided in Appendix A Table A2.

VOCs data were analyzed with R (version 3.6.3, Vienna, Austria) [27] in RStudio (version 1.2.5033, Boston, MA, USA) [28,29]. Firstly, the VOCs table was adjusted as follows: only compounds observed in at least 25% of samples were kept, natural log transformation was performed using the log() function and missing values were imputed to 0. Generalized linear mixed-effects, glmer() function of the lme4 package [30], was used to perform a mixed effect regression model to assess whether there was correlation between the number of VOCs and postnatal baby age (days). Finally, LME model analysis was performed with the lmer() function of the lme4 package [30]. Patients ID was used as a random factor, while baby age (days), GC-MS run batch, gestational age and delivery mode were the fixed factors. ggplot2 [31] package was used to produce the charts.

## 5. Conclusions

This study shows the evolution of the metabolome in early life in pre-term babies. We observed a clear shift in the metabolome after 5 days from birth that coincides with the establishment of enteral feeding and the transition from meconium to faeces.

## Figures and Tables

**Table 1:** Summary of basic demographic features and sampling from 51 preterm babies.

	Median	Range
Gestational age (weeks)	29	23–31 + 6 d
Birthweight (g)	1095	585–1820
Samples per donor	3	2–6

d: days.

**Table 2:** Summary of 36 volatile organic compounds (VOCs) found in at least 25% of samples.

**Short Chain Fatty Acids**	**Branched Chain Fatty Acids**	**Methylated Aldehydes**	**Esters**
Acetic acid	2-methylbutanoic acid	Isovaleraldehyde	Ethyl acetate
Propionic acid	Isovaleric acid	2-methylbutyraldehyde	Propyl acetate
Butanoic acid		Isobutyraldehyde	Ethyl propionate
			Propyl propionate
**Aldehydes**	**Alcohols**	**Ketones/Diketones**	**Others**
Hexanal	Ethanol	2-heptanone	2-ethylfuran
Heptanal	Propanol	4-heptanone	2-pentylfuran
Octanal	1-pentanol	6-methyl-5-hepten-2-one	D-limonene
Nonanal	1-hexanol	Acetoin	Methoxy-phenyl-oxime
Benzaldehyde	1-octen-3-ol	2,3-butanedione	1,4-xylene
Phenylacetaldehyde	2-ethylhexanol		Ethylbenzene

**Table 3:** Samples collected in each age group.

	Age Range (d)	Number Samples	Median Number of VOCs (Range)
Samples from R1	0–5	18	13 (6–22)
Samples from R2	6–10	44	17 (8–31)
Samples from R3	11–20	56	22.5 (8–30)
Samples from R4	21–70	34	24 (13–31)

VOCs: volatile organic compounds, d: days.

**Table 4:** List of volatile organic compounds (VOCs) that were influenced by relevant variables obtained with linear mixed-effects (LME) analysis.

Compound	Postnatal Age (Days)		Gestational Age (Weeks)		Delivery Mode	
	Slope	*p*-Value	Parameter	*p*-Value	Parameter	*p*-Value
**Short chain fatty acids**						
Butanoic acid	0.28	***	−	−	−	−
Acetic acid	0.08	*	−	−	−	−
Propionic acid	0.15	*	−	−	−	−
**Branched chain fatty acids**						
Isovaleric acid	0.2	**	−	−	−	−
2-methylbutanoic acid	0.24	***	−	−	−	−
**Esters**						
Ethyl acetate	0.19	**	0.87	*	−4.07	*
Propyl acetate	0.36	***	1.15	*	−	−
Ethyl propionate	0.27	***	0.88	*	−	−
Propyl propionate	0.36	***	1.02	*	−	−
**Aldehydes**						
Heptanal	0.11	*	−	−	−	−
Octanal	0.13	*	−	−	3.17	*
Nonanal	0.14	**	−	−	−	−
Benzaldehyde	0.15	**	−	−	−	−
Phenylacetaldehyde	0.2	***	−	−	−	−
**Alcohols**						
Propanol	0.38	***	−	−	−	−
1-octen-3-ol	−	−	−0.76	*	3.45	**
**Ketones/diketones**						
2-heptanone	−	−	−	−	3.95	**
4-heptanone	−	−	−1.38	***	−	−
6-methyl-5-hepten-2-one	0.18	***	−	−	−	−
Acetoin	0.18	**	−	−	−	−
2,3-butanedione	0.27	***	−	−	−	−
**Others**						
2-ethylfuran	−	−	−	−	2.66	*
2-pentylfuran	−	−	−	−	4.11	**
Methoxy-phenyl-oxime	0.15	**	−	−	−	−

A positive slope for infant age (days) indicates an increase in the compound over time; a positive value for gestational age indicates that babies born later had more of that compound; a positive value for delivery mode means that babies born through a caesarean section had more of that compound, opposite to a negative slope that refers to a compound being more prevalent in babies born by vaginal delivery. Values that were not significant are not shown (−). Significance codes: − *p* not significant, * *p* < 0.05, ** *p* <0.01, *** *p* < 0.001. All samples were included, n = 152.

## Data Availability

CDF files, VOCs and metadata tables are available upon reasonable request from the corresponding author.

## Sample Availability

Human samples are not available from the authors, in accordance with the Human Tissue Act, these have been used and remainder destroyed.

## Appendix A

**Table A1:** The prevalence of the 36 most abundant volatile in the four age groups.

	Prevalence in Each Age Group (%)				Mean Prevalence (%)
	G1	G2	G3	G4	
Acetic acid	88.9	86.4	92.9	94.1	90.8
Hexanal	100.0	86.4	91.1	82.4	88.8
Nonanal	66.7	68.2	87.5	94.1	80.9
Isovaleraldehyde	88.9	77.3	78.6	76.5	78.9
Heptanal	66.7	63.6	82.1	85.3	75.7
2,3-butanedione	11.1	63.6	85.7	94.1	72.4
Octanal	77.8	54.5	71.4	91.2	71.7
2-methylbutyraldehyde	66.7	63.6	73.2	70.6	69.1
4-heptanone	55.6	70.5	69.6	73.5	69.1
2-pentylfuran	72.2	61.4	69.6	73.5	68.4
Isovaleric acid	16.7	70.5	69.6	70.6	63.8
1-octen-3-ol	66.7	56.8	60.7	76.5	63.8
6-methyl-5-hepten-2-one	33.3	59.1	66.1	82.4	63.8
2-ethylhexanol	50.0	59.1	60.7	70.6	61.2
2-heptanone	66.7	59.1	62.5	52.9	59.9
Propanol	0.0	38.6	78.6	88.2	59.9
1-pentanol	61.1	54.5	51.8	64.7	56.6
2-methylbutanoic acid	16.7	61.4	50.0	73.5	54.6
Propionic acid	38.9	40.9	64.3	58.8	53.3
Acetoin	5.6	54.5	60.7	61.8	52.6
Benzaldehyde	38.9	45.5	42.9	76.5	50.7
Phenylacetaldehyde	11.1	38.6	55.4	70.6	48.7
Acetone	16.7	54.5	48.2	41.2	44.7
Butanoic acid	11.1	31.8	48.2	61.8	42.1
Propyl propionate	0.0	11.4	57.1	67.6	39.5
Methoxy-phenyl-oxime	44.4	27.3	37.5	52.9	38.8
Ethyl acetate	0.0	27.3	48.2	55.9	38.2
Propyl acetate	0.0	13.6	53.6	64.7	38.2
Ethanol	0.0	43.2	48.2	35.3	38.2
Isobutyraldehyde	16.7	36.4	37.5	47.1	36.8
Ethyl propionate	5.6	13.6	44.6	58.8	34.2
1,4-xylene	11.1	43.2	41.1	20.6	33.6
1-hexanol	27.8	38.6	33.9	29.4	33.6
D-limonene	0.0	22.7	39.3	47.1	31.6
Ethylbenzene	27.8	29.5	28.6	38.2	30.9
2-ethylfuran	33.3	25.0	25.0	26.5	26.3

**Table A2:** Names of compounds described as common names, IUPAC names and PubChem CID number.

Common Name	IUPAC Name	CID Number
Acetic acid	Acetic acid	176
Propionic acid	Propanoic acid	1032
Butanoic acid	Butanoic acid	264
2-methylbutanoic acid	2-methylbutanoic acid	8314
Isovaleric acid	3-methylbutanoic acid	10,430
Isovaleraldehyde	3-methylbutanal	11,552
2-methylbutyraldehyde	2-methylbutanal	7284
Isobutyraldehyde	2-methylpropanal	6561
Ethyl acetate	Ethyl acetate	8857
Propyl acetate	Propyl acetate	7997
Ethyl propionate	Ethyl propanoate	7749
Propyl propionate	Propyl propanoate	7803
Hexanal	Hexanal	6184
Heptanal	Heptanal	8130
Octanal	Octanal	454
Nonanal	Nonanal	31,289
Benzaldehyde	Benzaldehyde	240
Phenylacetaldehyde	2-phenylacetaldehyde	998
Ethanol	Ethanol	702
Propanol	Propan-1-ol	1031
1-pentanol	Pentan-1-ol	6276
1-hexanol	Hexan-1-ol	8103
1-octen-3-ol	Oct-1-en-3-ol	18,827
2-ethylhexanol	2-ethylhexan-1-ol	7720
Acetone	Propan-2-one	180
2-heptanone	Heptan-2-one	8051
4-heptanone	Heptan-4-one	31,246
6-methyl-5-hepten-2-one	6-methylhept-5-en-2-one	9862
Acetoin	3-hydroxybutan-2-one	179
2,3-butanedione	Butane-2,3-dione	650
2-ethylfuran	2-ethylfuran	18,554
2-pentylfuran	2-pentylfuran	19,602
D-limonene	(4*R*)-1-methyl-4-prop-1-en-2-ylcyclohexene	440,917
Methoxy-phenyl-oxime	methyl (*Z*)-*N*-hydroxybenzenecarboximidate	9,602,988
1,4-xylene	1,4-xylene	7809
Ethylbenzene	Ethylbenzene	7500
